# Use and efficacy of dry-mouth interventions in Sjögren’s disease patients and possible association with perceived oral dryness and patients’ discomfort

**DOI:** 10.1007/s00784-023-05172-2

**Published:** 2023-07-28

**Authors:** Z. Assy, J. S. van Santen, H. S. Brand, F. J. Bikker

**Affiliations:** Department of Oral Biochemistry, Academic Centre for Dentistry Amsterdam, University of Amsterdam and VU University Amsterdam, Amsterdam, The Netherlands

**Keywords:** Sjögren’s syndrome, Sjögren’s disease, Dry mouth, Xerostomia, Dry-mouth interventions, Saliva substitutes

## Abstract

**Objectives:**

Sjögren’s disease (SjD) patients use various interventions to relief their oral dryness. However, the use and efficacy of these interventions have only partially been evaluated. The present study aims to investigate whether there is an association between the perceived oral dryness and discomfort of SjD patients and their use of specific interventions.

**Materials and methods:**

A cross-sectional study was performed among SjD patients, who completed several questionnaires to assess the severity of their oral dryness and an inventory of dry-mouth interventions. The perceived efficacy of each intervention was reported on a 5-point Likert-scale.

**Results:**

The questionnaires were returned by 92 SjD patients. For relief of oral dryness, they mostly used “eating fruit”, “drinking tea”, “moistening the lips”, “drinking water, and “drinking small volumes” (> 50%). Three interventions had a frequency of use ranging from 2–6 times/day, whereas, “drinking water” and “drinking small volumes” showed higher frequencies (> 14). The highest overall efficacy (≥ 3.5) was reported for “chewing gum” and “using a mouth gel”. Furthermore, various dry-mouth interventions showed significant associations with oral dryness scores and/or patients’ discomfort. For example, “drinking small volumes” and “using XyliMelts” were associated with the Bother Index score.

**Conclusion:**

Great variation was found in the use of dry-mouth interventions by the participants and the severity of the oral dryness and/or patients’ discomfort seemed to affect their choice of intervention. Notably, the mostly used interventions did not show the highest reported efficacy.

**Clinical relevance:**

These findings might help SjD patients and clinicians in their choice of effective dry-mouth interventions.

**Supplementary Information:**

The online version contains supplementary material available at 10.1007/s00784-023-05172-2.

## Introduction

Sjögren’s disease (SjD) is a progressive autoimmune disease that affects, among others, the integrity of the exocrine lacrimal and salivary glands [1, 2]. Thus, SjD is associated with keratoconjunctivitis sicca, hyposalivation, and xerostomia [1]. Hyposalivation can induce negative (oral) consequences, such as an increased risk of oral infections, difficulties with swallowing and speaking, and sleep disturbances caused by xerostomia. These consequences can negatively affect the quality of life in general and oral health in particular [1, 3, 4].

In a recent study, it has been shown that SjD patients use a variety of different interventions/options to relieve their dry mouth complaints [5]. Generic, palliative dry-mouth interventions, such as “drinking water”, “moistening the lips”, “drinking tea”, and “rinsing of the mouth”, were mostly used. Although, less commonly, salivary stimulants were used by SjD patients. “Chewing gum” was the most commonly reported saliva stimulant, followed by “eating fruit” and “sucking sour candies”. In contrast, pharmaceuticals, such as pilocarpine, were used only by a limited number of SjD patients [5]. The various adverse effects of pilocarpine seem to contribute to its limited use: nausea, sweating, and headaches are commonly reported adverse events for individuals taking pilocarpine [3]. Additionally, systemic pharmaceuticals may be contraindicated in patients with comorbidity like chronic respiratory, cardiovascular, or renal diseases, limiting their use to relief dry-mouth complaints in a large group of patients [6].

The selection of particular interventions in SjD patients may be partially explained by the fact that the initial impairment mainly affects the submandibular and sublingual glands, leading to an increased presence of serous saliva and a decreased rate of unstimulated salivary flow. As the disease progresses; eventually, the parotid glands are generally affected [7]. Therefore, in the beginning of SjD, moistening of the intra-oral surfaces can still partially be achieved through mechanical stimulation of the parotid glands. However, later on, the function of all major salivary glands is severely impaired [5]. As a consequence, palliative care options, such as the use of saliva substitutes, remain to relief oral dryness. A wide range of salivary substitutes such as mouth sprays, gels, and washes are available and used by SjD patients. In addition to these artificial saliva’s, so-called XyliMelts have been investigated in a group of patients with various causes of oral dryness. XyliMelts are mucosal adhering disks that release xylitol and cellulose gum [8]. However, the perceived effectiveness of XyliMelts has not previously been evaluated in SjD patients.

So, various dry mouth interventions are being used by SjD patients [5]. However, their frequency of use and perceived efficacy have not systematically been investigated. For this reason, the present study aimed to investigate the use, frequency of use, and perceived efficacy of various dry-mouth interventions among SjD patients. Besides, it was investigated whether patients’ discomfort affects the choice of dry-mouth interventions. The results of this investigation could provide clinicians with information to give SjD patients a more targeted advice for the relief of dry-mouth complaints.

## Materials and method

### Study design

A cross-sectional study was performed among members of the Dutch Sjögren’s Patients Federation (Nationale Vereniging Sjögrenpatiënten, NVSP) who attended the annual meeting of this Federation in Breukelen, the Netherlands on October 1st, 2022. According to self-reported information from these members, they suffered from Sjögren’s disease. Volunteers could anonymously fill in a questionnaire and return it in a designated mailbox during the meeting or return the questionnaire by mail using an enclosed prepaid envelope. The local Ethics Review Committee of the Academic Center for Dentistry Amsterdam (ACTA) confirmed that the Medical Research Involving Human Subjects Act (WMO) did not apply to this study (protocol number 201930). The reporting of this study conforms to the STrengthening the Reporting of OBservational studies in Epidemiology (STROBE) statement [9].

### Study variables

The questionnaire, developed for this study, consisted of five parts. The first part consisted of some general questions with regard to age, sex, and a question in which year Sjögren’s disease had been diagnosed by a rheumatologist. Second, the internationally validated xerostomia inventory (XI) was used to measure the overall oral dryness of the patients [10]. The XI consists of 11 items on a 5-point Likert scale ranging from 1—“Never” to 5—“Very often”. The items concern patients’ oral dryness and feeling of the mouth, for example, “my mouth feels dry when eating a meal”. For each item, patients indicate how often they experience the feeling of dryness in the mouth and problems regarding oral dryness. The scores of the 11 items are summed to produce a total XI-score that ranges between 11 (no xerostomia) and 55 (extreme xerostomia) [10]. The third part of the questionnaire comprised of the bother index (BI). In the BI, the patient was asked to rate the severity of dry mouth on a scale from 0 to 10 [11–15]. The patients could also indicate which moment of the day they were mostly affected by a dry mouth: morning, afternoon, evening, or at night. The fourth part of the questionnaire was the Regional Oral Dryness Inventory (RODI), which determines differences in dry mouth perception at different intra-oral regions. The RODI questionnaire contains nine schematic illustrations of different locations in the oral cavity [16, 17]. Four illustrations represent areas in the upper jaw: the upper lip, the posterior part of the palate (from the rugae up to the end of the soft palate), the anterior part of the palate (including the rugae), and the inside part of the cheeks. Four other illustrations represent areas in the lower jaw: the lower lip, the anterior part of the tongue (from the tip of the tongue up to the vallate papilla), the posterior part of the tongue (from the vallate papilla up to the end of the tongue), and the floor of the mouth. Finally, one illustration represents the pharynx. At each location, the patient can indicate the severity of the intra-oral dryness on a 5-point Likert scale ranging from 1—“No dryness” to 5—“Severe dryness” [16, 17]. The questionnaire concluded with a list of potential interventions to relief the feeling of a dry mouth [5, 18] (Table 3). “Drinking small volumes” was specifically added to highlight the conscious decision when using liquid dry-mouth interventions. This refers to the distinction between regular drinking, for example, drinking water at once, or a conscious decision to use small sips. For each intervention, a participant could report whether or not they used the intervention, and if they did, what the frequency of use was throughout the day. Besides,they could indicate how effective they perceived the intervention to be.

SjD﻿ ﻿patients who actively checked "YES” for the question on the current use of an intervention were considered as current users. Patients who used an intervention method in the past could, however, also fill in how they had perceived the efficacy of that intervention, leading to an overall assessment of the efficacy of each intervention method. The efficacy of each intervention was questioned by a 5-point Likert scale, ranging from 1—“Not effective” to 5—“Highly effective”. To reflect on the perceived efficacy by the current users, the number of current users who indicated an efficacy of ≥ 4 (effective or highly effective) for each intervention was also reported. If a participant indicated to use the intervention “throughout the day” instead of a specific number; then, the highest number reported by other participants for this intervention was chosen. For “rinsing of the mouth”, participants could also indicate which liquid was used for rinsing. For “using a mouth gel”, participants had to indicate where they apply the mouth gel, using the same regions of the RODI as described above. In addition, participants were asked whether they used any odors, which could be any smell, perfume, or scent, to relief their perceived oral dryness, and if so, which odor they used for this purpose.

### Data analysis

The data were statistically analyzed with SPSS, version 27.0 (IBM Corp SPSS statistics, Armonk, NY, USA). The Shapiro–Wilk test was used to assess the normality of the data. As not all variables were normally distributed, the data are presented as medians and their interquartile range (IQR) and/or as the mean and standard deviation (SD). Because most of the data were not normally distributed, non-parametric tests had been used for all statistical analysis. The questionnaires were not filled in completely by all volunteers; therefore, the total number (N) could vary.

A Friedman test was conducted for the RODI scores of the study sample, followed by a Wilcoxon signed rank test as a post hoc procedure. The differences in the efficacy of the five most commonly used interventions by the participants were investigated using the Friedman test, followed by a Wilcoxon signed rank test as a post-hoc procedure.

The association between the mostly used interventions (> 50% of participants) with oral dryness (XI and RODI scores) and patients’ discomfort (BI scores) was initially investigated with a binary logistic regression. Each dry-mouth intervention was considered as a dependent variable, and the total XI, BI, and RODI scores of the nine intra-oral regions were considered as independent variables. To identify the degree of multicollinearity among the independent variables, the variance inflation factor (VIF) was calculated. The VIF for these variables was < 5, which indicates that there was no multicollinearity present among these variables [18, 19], so they do not influence each other. The backward conditional method was used to analyze these independent variables. If there was a significant association between a dry-mouth intervention and one or more independent variables; then, the odds ratio (OR) and the 95% confidence interval (95% CI) were reported. Furthermore, the last step of the Omnibus test chi-square and the Hosmer and Lemeshow (H–L) test chi-square including their degree of freedom (df) and their *p* values were reported. Also, the Coxx-Snell R square and the Nagelkerke R square were mentioned, if the association was significant.

Further analysis was done to assess the possible association between mostly used interventions (> 50%) among participants and the most dry regions of the RODI specifically. A Mann–Whitney *U* tests was used to investigate whether these regions affected the use of the mostly used interventions.

All significance levels (α) were set at 0.05.

## Results

At the 2022 annual meeting of the SjD patient federation, 164 questionnaires were distributed. In total, 92 questionnaires were returned, resulting in a 56.7% response rate. Most of the respondents were female (*N* = 78, 84.8%), and 3 of the respondents were male (3.3%). 11 respondents did not report their sex (12%). The mean age of the respondents was 64 ± 11 years, ranging from 30 to 88 years. Almost all patients (*N* = 83, 90.2%) indicated that their SjD diagnosis was confirmed by a rheumatologist. The year when, according to the patients, SjD was diagnosed ranged from 1958 to 2022.

### Perceived oral dryness and patients’ oral discomfort

The overall dry-mouth feeling was quantified with the XI. The mean total XI score of the study sample was 44.7 ± 6.6 with a median score of 45.0 and IQR of 41.0–50.0 (*N* = 90). Patients’ dry-mouth discomfort as measured with BI had a mean of 7.5 ± 1.9 with a median score of 8.0 and IQR of 7.0–9.0 (*N* = 88). Most of the patients reported having discomfort mostly at night (82.6%) and in the morning (75.0%). Patients’ discomfort was less during the afternoon (62%) and in the evening (63%).

The perceived oral dryness at various intra-oral locations, as determined by the RODI questionnaire, is presented in Table 1. Perceived oral dryness in the study sample differed significantly among the nine intra-oral regions (Friedman test *p* < 0.05, followed by Wilcoxon signed-rank test). It was found that the perceived intra-oral dryness was most severe for the pharynx and the posterior palate. In contrast, the inside of the cheeks was experienced as the least dry. The pharynx had significant differences with all intra-oral regions, except for the posterior palate.

**Table 1:** Perceived oral dryness of SjD patients at nine intra-oral regions as determined with the RODI. Data are presented as median with corresponding IQR and as a mean with SD. *N* indicates the total number of respondents for each intra-oral region

Intra-oral regions	Mean	SD	Median	IQR	*N*
Upper lip	3.2	1.1	3.0	2.0–4.0	91
Inside cheeks	3.1	1.1	3.0	3.0–4.0	88
Anterior palate	3.2	1.2	3.0	3.0–4.0	89
Posterior palate^a,b,c^	3.8	1.0	4.0	3.0–4.0	91
Lower lip^d^	3.2	1.1	3.0	2.0–4.0	88
Floor of the mouth^d^	3.2	1.1	3.0	3.0–4.0	89
Anterior tongue^a,b,c,d,e,f^	3.5	1.2	3.5	3.0–4.0	86
Posterior tongue^a,b,c,d,e,f^	3.6	1.0	4.0	3.0–4.0	89
Pharynx^a,b,c,e,f,g,h^	3.9	1.0	4.0	3.0–5.0	88

^a^Wilcoxon signed-rank tests: *p* < 0.05 vs. upper lip. ^b^Wilcoxon signed-rank tests: *p* < 0.05 vs. inside cheeks. ^c^Wilcoxon signed-rank tests: *p* < 0.05 vs. anterior palate. ^d^Wilcoxon signed-rank tests: *p* < 0.05 vs. posterior palate. ^e^Wilcoxon signed-rank tests: *p* < 0.05 vs. lower lip. ^f^Wilcoxon signed-rank tests: *p* < 0.05 vs. floor of the mouth. ^g^Wilcoxon signed-rank tests: *p* < 0.05 vs. anterior tongue. ^h^Wilcoxon signed-rank tests: *p* < 0.05 vs. posterior tongue

### Interventions to relief perceived dry mouth: use and frequency

Most of the SjD patients used, or previously used, multiple interventions to relief their perceived oral dryness (Table 2). Only a limited number of patients used no or a single intervention (2.2%). The majority of the patients used 6–9 different interventions for relief of their dry mouth (64.2%). One participant even used ≥ 15 different interventions to relief oral dryness.

**Table 2:** Number of different dry-mouth interventions used by SjD patients. Data are presented as by the number of patients and percentages. *N* = 87

No. of dry-mouth interventions	No. of participants (%)
0–1	2 (2.2)
2–5	10 (11.5)
6–9	56 (64.2)
10–14	18 (20.7)
≥ 15	1 (1.1)

Table 3 gives an overview of all interventions, as used by SjD patients, and the frequency of the use per day of the applied interventions. The interventions mostly used (by > 50% of participants) for the relief of oral dryness were “drinking water”, “eating fruit”, “drinking tea”, “moistening the lips”, “drinking small volumes”, “drinking coffee”, and “rinsing of the mouth”. Other interventions such as “drinking soft drinks”, “eating cucumber with olive oil during meal”, “drinking lemonade”, “drinking beer”, “putting olive oil in the mouth”, “using pilocarpine”, “putting lemon slices in the mouth”, and “sucking ice cubes” were less prevalent among SjD patients. Over 50% of the patients indicated to rinse their mouth as an intervention. “Water” was the most reported liquid used for rinsing, while 3 participants indicated that they used a “mouth wash” for rinsing.

**Table 3:** Use of interventions by SjD patients for the relief of dry-mouth symptoms. For each intervention, the percentage of participants currently using the intervention is reported, as well as the frequency (times/day) and overall perceived efficacy, based on a 5-point Likert scale. All data are presented as mean ± SD. In addition, the number and percentage of current users, who reported high efficacy (≥ 4), are included

Dry-mouth interventions	Percentage of participants currently using the intervention (%)	Frequency times/day (Mean ± SD)	Overall perceived efficacy 5-point Likert scale (Mean ± SD)	Efficacy ≥ 4 reported by current users *N* (%)
Drinking water	81.5	15.7 ± 11.0	3.2 ± 1.0	38 (50.7%)
Eating fruit	77.2	1.8 ± 0.8	2.9 ± 1.2^a^	21 (29.6%)
Drinking tea	75.0	4.8 ± 5.8	2.8 ± 1.1^a^	24 (34.8%)
Moistening the lips	66.3	6.0 ± 7.1	3.4 ± 1.1^b,c^	37 (60.7%)
Drinking small volumes	64.1	25.3 ± 29.2	3.2 ± 1.1	29 (49.2%)
Drinking coffee	64.1	3.3 ± 4.3	2.5 ± 1.1^a,c,d,e^	13 (22.0%)
Rinsing of the mouth	52.2	5.7 ± 6.6	3.0 ± 1.1	17 (35.4%)
Concentrating on other activities	48.9	14.0 ± 7.2	3.1 ± 1.1	14 (31.1%)
Chewing gum	37.0	3.5 ± 4.5	3.6 ± 1.4	28 (82.4%)
Sucking sour candies	33.7	3.6 ± 4.7	3.2 ± 1.2	19 (61.3%)
Using XyliMelts	33.7	1.7 ± 1.2	3.4 ± 1.4	21 (67.7%)
Using mouth gel	33.7	1.9 ± 1.1	3.5 ± 1.1	21 (67.7%)
Using mouth spray	18.5	2.6 ± 1.5	2.8 ± 1.4	10 (58.8%)
Drinking soft drinks	17.4	4.6 ± 8.3	2.1 ± 1.2	5 (31.3%)
Eating cucumber with olive oil during meal	16.3	1.2 ± 0.8	2.9 ± 1.5	9 (60.0%)
Drinking lemonade	15.2	4.8 ± 8.2	2.3 ± 1.3	7 (50.0%)
Drinking beer	8.7	0.7 ± 0.7	2.1 ± 1.3	4 (50.0%)
Putting olive oil in the mouth	7.6	1.3 ± 1.5	2.2 ± 1.6	3 (42.9%)
Using pilocarpine	6.5	2.4 ± 1.7	2.3 ± 1.6	2 (33.3%)
Putting lemon slices in the mouth	4.3	NR	2.3 ± 1.6	4 (100%)
Sucking ice cubes	4.3	6.8 ± 11.5	1.9 ± 1.2	3 (75.0%)
Other interventions	10.9	NR	NR	NR

^a^Wilcoxon signed-rank tests: *p* < 0.05 vs. drinking water. ^b^Wilcoxon signed-rank tests: *p* < 0.05 vs. eating fruit. ^c^Wilcoxon signed-rank tests: *p* < 0.05 vs. drinking tea. ^d^Wilcoxon signed-rank tests: *p* < 0.05 vs. moistening the lips. ^e^Wilcoxon signed-rank tests: *p* < 0.05 vs. drinking small volumes. *NR*, not reported

For patients who indicated that they used a mouth gel for relief of their oral dryness, the most frequently reported regions where the mouth gel was applied were the anterior part of the tongue (23.9%), inside cheeks (21.7%), and posterior part of the tongue (19.6%). The mouth gel was less frequently applied to all other intra-oral regions, with percentages ranging between 12% for the upper and lower lip to 16.3% for the posterior part of the palate.

The frequency of use per day was the highest for “drinking water”, “drinking small volumes”, and “concentrating on other activities”, indicating that patients used these interventions multiple times a day (≥ 14 times/day). All other interventions were used between 1–7 times/day (Table 3).

The spontaneously reported “using other interventions” included “using mint flavored lozenges”, “using mouth wash”, “using specialized toothpaste”, and “eating cucumber”. No frequencies and efficacies were reported in this subgroup.

### Dry-mouth interventions: efficacy

Table 3 reports the efficacy of the interventions used to reduce perceived dry-mouth feeling. The highest efficacy (≥ 3.5) was reported for “chewing gum” and “using a mouth gel”. The efficacy for the majority of the other interventions ranged between 1.9 and 2.9, indicating that most of the interventions were not found to be effective for the relief of a dry mouth. Only a few interventions were reported to have a moderate effectivity (≥ 3.0 efficacy < 3.5); “drinking water”, “moistening the lips”, “drinking small volumes”, “rinsing of the mouth”, “concentrating on other activities”, “sucking sour candies”, and “using XyliMelts”.

The efficacy of the top five interventions differed significantly (Friedman test *p* < 0.01). “Drinking coffee” was a less effective intervention compared to almost all other interventions (Wilcoxon signed-rank test *p* < 0.05). “Moistening the lips” was reported to have the highest efficacy of the top five interventions and differed significantly from “eating fruit” and “drinking tea”. Furthermore, no significant difference in efficacy was found between “drinking water”, “moistening the lips”, and “drinking small volumes” (Wilcoxon signed-rank test *p* > 0.05), suggesting that these three interventions can be considered equally effective in their ability to relief perceived oral dryness.

Table 3 also reports the number of current users who indicated an efficacy of ≥ 4 (effective to highly effective). Highly effective interventions are “putting lemon slices in the mouth”, “chewing gum”, “sucking ice cubes”, “using XyliMelts”, and “using a mouth gel” (> 67% of the SjD patients using these interventions). This result is in line with the overall efficacy for “chewing gum”, “using a mouth gel”, and “using XyliMelts”, which also had the highest overall perceived efficacy. However, for “putting lemon slices in the mouth” and “sucking ice cubes”, the overall perceived efficacy ranged between 1.9 and 2.3. For the other interventions, the percentage of users reporting an efficacy of ≥ 4 ranged between 22.0 and 61.3%, indicating that these interventions were less effective for relieving perceived oral dryness.

### Association between the use of dry-mouth interventions and perceived oral dryness

The association between the use of dry-mouth interventions and perceived oral dryness was further investigated. Severe perceived dryness of the posterior palate and the pharynx (RODI score ≥ 4) showed no association with the use of any dry mouth intervention (Mann–Whitney *U* test, *p* > 0.05, Supplementary file S1).

A binary logistic regression was performed to investigate whether perceived oral dryness and/or patients’ discomfort was associated with the use of the more mostly used dry-mouth interventions. In Table 4, the odds ratios for the dry-mouth interventions are reported. Interventions such as “drinking water”, “drinking tea”, “drinking coffee”, and “using mouth gel” did not have any significant association with any of the included independent variables; for this reason, these interventions are not included in Table 4. The use of “drinking small volumes”, “rinsing the mouth”, and “using XyliMelts” were all significantly associated with the BI scores of the users. The odds ratio varied between 1.45 and 2.85, indicating that higher BI scores (increased patients’ discomfort) were associated with an increased likelihood of using these interventions. “Moistening of the lips” was significantly associated with the total XI score (OR = 1.1); thus, higher XI scores (severe overall mouth dryness) increase the likelihood of use of this specific intervention.

**Table 4:** The odds ratio of several independent variables (BI, total XI, and RODI scores) for the mostly used interventions (> 50%) by current users. The odds ratio including the 95% CI is reported. For the significant associations also, the last step of the Omnibus and H–L test Chi-square including their df and *p* values was reported. Furthermore, the Coxx-Snell and the Nagelkerke R square were mentioned

	Eating fruit	Moistening the lips	Drinking small volumes	Rinsing of the mouth	Concentrating on other activities	Chewing gum	Sucking sour candies	Using XyliMelts
BI-score	NS	NS	2.85 (1.35–6.03)^**,c^	1.45 (1.06–2.00)^*,d^	NS	NS	NS	1.73 (1.14–2.62)^**,h^
Total XI-score	NS	1.11 (1.02–1.21)^*,b^	NS	NS	NS	NS	NS	NS
RODI-score of upper lip	NS	NS	0.21 (0.05–0.91)^*,c^	NS	NS	NS	NS	NS
RODI-score of anterior palate	NS	NS	4.10 (1.10–15.24)^*,c^	NS	NS	NS	NS	NS
RODI-score of lower lip	NS	NS	NS	NS	1.96 (1.18–3.25)^**, e^	0.36 (0.19–0.70)^**,f^	NS	NS
RODI-score of anterior tongue	NS	NS	NS	NS	NS	NS	0.39 (0.17–0.88)^*,g^	NS
RODI-score of posterior tongue	3.58 (1.08–11.80)^*,a^	NS	0.24 (0.06–0.96)^*,c^	NS	NS	NS	NS	NS

*NS*, the independent variable was not significant. Binary logistic regression: ^*^p < 0.05. Binary logistic regression: ^**^p < 0.01. ^a^H-L test χ^2^ = 12.0, df = 8, *p* > 0.05; Omnibus test χ^2^ = 7.0, df = 3, *p* > 0.05; Cox-Snell *R*^2^ = 0.09; Nagelkerke *R*^2^ = 0.16. ^b^H-L test χ^2^ = 9.2, df = 8, *p* > 0.05; Omnibus test χ^2^ = 6.2, df = 1, *p* < 0.05; Cox-Snell *R*^2^ = 0.08; Nagelkerke *R*^2^ = 0.13. ^c^ H–L test χ^2^ = 6.8, df = 8, *p* > 0.05; Omnibus test χ^2^ = 18.2, df = 6, *p* < 0.01; Cox-Snell *R*^2^ = 0.25; Nagelkerke *R*^2^ = 0.40. ^d^H-L test χ^2^ = 2.5, df = 4, *p* > 0.05; Omnibus test χ^2^ = 6.5, df = 1, *p* < 0.05; Cox-Snell *R*^2^ = 0.09; Nagelkerke *R*^2^ = 0.12. ^e^H-L test χ^2^ = 8.9, df = 8, *p* > 0.05; Omnibus test χ^2^ = 8.4, df = 2, *p* < 0.05; Cox-Snell *R*^2^ = 0.11; Nagelkerke *R*^2^ = 0.15. ^f^H-L test χ^2^ = 8.9, df = 7, *p* > 0.05; Omnibus test χ^2^ = 11.3, df = 2, *p* < 0.01; Cox-Snell *R*^2^ = 0.15; Nagelkerke *R*^2^ = 0.20. ^g^H-L test χ^2^ = 5.6, df = 8, *p* > 0.05; Omnibus test χ^2^ = 11.1, df = 4, *p* < 0.05; Cox-Snell *R*^2^ = 0.15; Nagelkerke *R*^2^ = 0.21. ^h^H-L test χ^2^ = 3.4, df = 4, *p* > 0.05; Omnibus test χ^2^ = 9.8, df = 1, *p* < 0.01; Cox-Snell *R*^2^ = 0.13; Nagelkerke *R*^2^ = 0.17

Various dry-mouth interventions were significantly associated with various regions of the RODI questionnaire (Table 4). None of the most commonly used interventions were significantly associated with the RODI scores of inside cheeks, posterior palate, floor of the mouth, and pharynx. As a result, these particular regions are not included in Table 4. Notably, even the most dry regions, the posterior palate, and the pharynx, did not have any significant association. However, “drinking small volumes” was significantly associated with the RODI score of the anterior palate (OR > 4.0), which indicates that severe dryness at the palate increased the likelihood to use this intervention. “Concentrating on other activities” was significantly associated with the RODI scores of the lower lip, while “eating fruit” had an OR = 3.6 with the RODI score for the posterior tongue. Otherwise, some interventions reported in Table 4 had an OR < 1, which indicated that SjD patients with less dry intra-oral regions (low RODI scores) are more likely to use this specific dry-mouth intervention.

## Discussion

The results of this cross-sectional study present an overview of the frequency of use and respective perceived effectivity of various dry-mouth interventions, i.e., options to relieve complaints of oral dryness, used by a group of 92 Dutch SjD patients. In general, great variation was found in the use of dry-mouth interventions. The severity of the perceived oral dryness and/or patients’ discomfort seemed to affect their choice of intervention. It has to be noted that, due to ethical and privacy issues, we were unable to obtain a confirmed diagnosis for SjD by a medical specialist of the participants. This unavoidable limitation of the study might have introduced some bias [19–21].

The majority of SjD patients in this study (86%) used, or previously used, 6 or more different dry-mouth interventions. In a previous study with a comparable character, it was reported that 55% of the participants used 4 or more products to relief oral dryness [20]. This difference could be related to the fact that in the previous study, dry-mouth patients with different etiologies were included. In the present study, solely participants with an SjD background were included, while in the study of Purdie and co-workers, a xerostomia cohort was included with a range of potential etiologies of which 54% were SjD patients [19]. In addition, in the present study, 21 different interventions were evaluated, whereas Purdie et al. only evaluated 8 methods, namely: “water”, “gum”, “lozenge”, “candies”, “rinses”, “sprays”, “gels”, and “parasympathomimetic medication” [20], explaining the higher numbers of applied interventions in the present study.

“Drinking water” was found as the mostly used intervention by participants, which is in line with the findings of Purdie et al. [20]. Obviously, in Western countries, water is highly accessible and is therefore a very easy option. For “drinking water” and “drinking small volumes”, their high frequencies could be explained by the short working effects and lack of lubricating properties in contrast to saliva, which is contained with, e.g. mucins, which aid in the retention of fluids in the oral cavity [22, 23]. Other frequently used interventions were “eating fruit”, “moistening the lips”, and “concentrating on other activities” were found to be applied frequently. “Eating fruit” was used by 77.2% of the study participants in the current study, as opposed to 40.5% in the previous study [5]. We speculate that this increase in “eating fruit” is related to the fact that this study was conducted in the late phase of the COVID-19 pandemic. The pandemic apparently nudged people to increase their consumption of fresh fruits and vegetables [24, 25]. Moreover, many fruits are known for their acidic taste which leads to sialagogic effects [26].

Besides, “concentrating on other activities” was frequently reported (≥ 14 times/day) this can be explained by the patients’ BI scores, which indicated that they experienced discomfort throughout the day. Therefore, the practice of “concentrating on other activities” would have to be applied frequently, helping to divert their thoughts and minimalize their dry-mouth complaints, which is supported by others showing that focusing on other external foci reduced discomfort, fatigue, and pain [27, 28].

With regard to efficiency, almost all commonly used interventions showed a moderate result (± 3.0). The highest overall efficacies (≥ 3.5) were observed for “chewing gum” and “using a mouth gel”. In turn, Purdie et al. reported a limited efficiency for “chewing gum”, as well as for “rinses”, “gel”, “spray”, and “candies” [20]. These investigators also reported that parasympathomimetic drugs such as pilocarpine had the highest perceived efficacy. Yet, in our study, the saliva secretion rates were not measured, rendering a comparison of the efficacy of pilocarpine with the therapies we studied difficult.

An interesting observation is that interventions that were most frequently used and/or most commonly used by participants did not perse have the highest perceived efficacy. In fact, less commonly used interventions, such as “putting lemon slices in the mouth”, “chewing gum”, “using XyliMelts”, and “using a mouth gel”, were considered the most effective. Notably, mouth gels are used as an intervention for the improvement of other oral problems such as a burning mouth, difficulty with mastication, and swallowing too [29]. Despite its high effectivity, the “use of a mouth gel” was not even used by half of the SjD patients in our study group. This might be attributed to the fact that over-the-counter medications for the relief of oral dryness, such as mouth gels and XyliMelts, usually are not reimbursed by health insurance companies in the Netherlands. In addition, a previous study has shown that the consistency and taste of most mouth gels are not appreciated by SjD patients [30]. “Putting lemon slices in the mouth” does not seem to be recommended SjD patients still having their dentition. The use of lemon slices results in a low intra-oral pH, especially in patients with dry mouth, which increases the risk of developing dental erosion. Previous research has shown that XyliMelts improved the perceived intra-oral wetness in patients with different causes various causes of oral dryness [8]. Our results indicate that XyliMelts have a similar positive effect in SjD patients (Table 3).

Recently, we investigated the associations between perceived oral dryness scores and the use of some dry-mouth interventions. In brief, that study encompassed an inventory of dry mouth interventions in SjD patients with no focus on their frequency of use and perceived efficacy [5]. So to some extent, both studies share some characteristics. Yet, in turn, differences are found with regard to goal, study design, and study population, which might lead to some differences in outcome. Therefore, some prudency should be taken to generalize these findings to the general population. For example, although both study groups contained dry-mouth patients with an SjD background, the identity and related background knowledge and experience with dry-mouth interventions of the volunteers in both groups probably deviated. Members of a patient federation might be better informed about the state-of-the-art of current SjD research and current intervention methods. Also, the socio-economical status of the participants could affect the use of interventions, where people with fewer financial resources possibly choose cheaper interventions for the relief of oral dryness. For ethical purposes, the identity of the volunteers could not be disclosed. Next, in contrast to the previous study, the current study included a questionnaire with yes/no items to limit acquiescence bias [31]. Besides, the current study included questions on “using XyliMelts”, “eating cucumber with olive oil during meal”, and “concentrating on other activities” and found an association between “moistening the lips” and the XI score. Hence, the studies deviated in the association between perceived oral dryness and “drinking tea” and no association was found with the use of any of the dry-mouth interventions and the pharynx and the posterior palate (Table S1) [5].

A potential limitation of the current study is that objective loss of saliva production (salivary flow rate) was not assessed. In the future, to more thoroughly investigate the effects of interventions with high perceived efficacy, a prospective controlled study would be advised. It would then be possible to include SjD patients who do not use the intervention of interest, to then be instructed to start using it and evaluate its efficacy over time. These findings could elucidate the relation between the effectiveness of the intervention and the location of the oral dryness. It would also be important to address the salivary secretion rate and as the disease progresses to discern between the differences in gland function, to increase the resolution of the outcomes and subsequent clinical value.

## Main conclusions

All in all, there is great variation in the use of dry-mouth interventions by SjD patients. The most commonly used interventions did not show the highest efficacy, whereas the severity of the oral dryness and/or patients’ discomfort affected which intervention was used. These findings could provide SjD patients and their clinicians with more insight into which intervention is most effective for the relief of dry-mouth complaints.

## Supplementary Information

Below is the link to the electronic supplementary material.

Supplementary file1 (DOCX 15 KB)

## Data Availability

Data available on request from the authors.
