# Supplementary material for: Use and efficacy of dry-mouth interventions in Sjögren’s disease patients and possible association with perceived oral dryness and patients’ discomfort
Source: Clin Oral Investig. 2023 Jul 28;27(9):5529–37. doi: 10.1007/s00784-023-05172-2 (PMC10492750; doi:10.1007/s00784-023-05172-2)
Supplement: Supplementary file 1 — Supplementary file1 (DOCX 15 KB) [file 784_2023_5172_MOESM1_ESM.docx]

**Supplementary data**

*Table S1: The RODI scores for the posterior palate and the pharynx, stratified based on the use of all mostly used interventions (>50%). Data are presented as mean ± SD.*

| More prevalent methods | Use | Posterior palate | Pharynx |
| --- | --- | --- | --- |
| Drinking water | Yes  No | 3.85 ± 0.97  3.67 ± 1.53 | 3.92 ± 1.00  2.00 |
| Eating fruit | Yes  No | 3.80 ± 1.02  4.00 ± 0.89 | 3.77 ± 1.06  4.18 ± 0.87 |
| Drinking tea | Yes  No | 3.81 ± 1.03  4.00 ± 0.71 | 3.87 ± 1.06  3.67 ± 1.00 |
| Moistening the lips | Yes  No | 3.89 ± 1.02  3.65 ± 0.93 | 3.93 ± 1.03  3.44 ± 0.98 |
| Drinking small volumes | Yes  No | 3.80 ± 1.00  3.73 ± 1.10 | 3.86 ± 0.98  3.57 ± 1.28 |
| Drinking coffee | Yes  No | 3.76 ± 1.02  4.00 ± 0.92 | 3.78 ± 1.06  4.00 ± 1.00 |
| Rinsing of the mouth | Yes  No | 3.85 ± 1.01  3.79 ± 0.99 | 3.89 ± 1.01  3.84 ± 1.02 |
| Concentrating on other activities | Yes  No | 3.80 ± 0.87  3.84 ± 1.14 | 3.95 ± 0.91  3.64 ± 1.15 |
| Chewing gum | Yes  No | 3.76 ± 0.92  3.85 ± 1.05 | 3.72 ± 0.96  3.91 ± 1.09 |
| Sucking sour candies | Yes  No | 3.71 ± 1.10  3.87 ± 0.92 | 3.73 ± 1.11  3.85 ± 0.99 |
| Using XyliMelts | Yes  No | 3.97 ± 0.91  3.75 ± 1.05 | 3.84 ± 1.00  3.82 ± 1.06 |
| Using mouth gel | Yes  No | 3.97 ± 0.98  3.73 ± 1.02 | 3.93 ± 0.91  3.70 ± 1.09 |
